# Protein kinase A signaling and calcium ions are major players in PAF mediated toxicity against *Aspergillus niger*

**DOI:** 10.1016/j.febslet.2015.03.037

**Published:** 2015-05-08

**Authors:** Ulrike Binder, Mojca Benčina, Ádám Fizil, Gyula Batta, Anil K. Chhillar, Florentine Marx

**Affiliations:** a Biocenter, Division of Molecular Biology, Medical University of Innsbruck, Innrain 80, A-6020 Innsbruck, Austria; b Division of Hygiene and Medical Microbiology, Schöpfstrasse 41, Medical University of Innsbruck, A-6020 Innsbruck, Austria; c Department of Biotechnology, National Institute of Chemistry, Hajdrihova 19, SI-1000 Ljubljana, Slovenia; d Department of Organic Chemistry, Faculty of Science and Technology, University of Debrecen, Egyetem tér 1, H-4032 Debrecen, Hungary; e Centre for Biotechnology, Maharshi Dayanand University, IN-124001 Rohtak, Haryana, India

**Keywords:** Antifungal protein, PAF, *Aspergillus niger*, Calcium homeostasis, Protein kinase A signaling

## Abstract

The *Penicillium chrysogenum* antifungal protein PAF is toxic against potentially pathogenic Ascomycetes. We used the highly sensitive aequorin-expressing model *Aspergillus niger* to identify a defined change in cytoplasmic free Ca^2+^ dynamics in response to PAF. This Ca^2+^ signature depended on an intact positively charged lysine-rich PAF motif. By combining Ca^2+^ measurements in *A. niger* mutants with deregulated cAMP/protein kinase A (PKA) signaling, we proved the interconnection of Ca^2+^ perturbation and cAMP/PKA signaling in the mechanistic function of PAF. A deep understanding of the mode of action of PAF is an invaluable prerequisite for its future application as new antifungal drug.

## Highlights

• *Aspergillus niger* is highly susceptible to the antifungal protein PAF.
• Ca^2+^ and cAMP/PKA signalling are interconnected in response to PAF.
• PAF toxicity requires the activation of the cAMP/PkaA signaling cascade.
• PAF evokes a sustained increase in the [Ca^2+^]_c_ resting level.
• Only a functional PAF protein interferes with the fungal Ca^2+^ homeostasis.
• PAF represents a promising molecule to develop new antifungal strategies.

## 1 Background

The number of newly identified small, cationic, cysteine-rich antifungal proteins that are produced by filamentous Ascomycetes is steadily increasing. Our knowledge about their mode of action, however, badly lacks behind, although scientists put major efforts into their characterization. This phenomenon may be based on variable degrees in similarity concerning primary sequence, solution structure, conformational dynamics, mechanistic function and antifungal spectrum [1–5]. However, most of these antifungal proteins are toxic against human-, animal- and plant pathogenic filamentous fungi, but less or not effective against bacteria or yeasts [6]. One of the best-studied antifungal proteins is PAF from *Penicillium chrysogenum* that elicits a complex response and ultimately triggers programmed cell death in sensitive target fungi (reviewed in [2]). Our previous studies indicated that the activation of signaling cascades, such as the cAMP/protein kinase A (PKA) signaling pathway, mediate the growth inhibitory activity of PAF in the model fungus *Aspergillus nidulans* [7]. Furthermore, a severe perturbation of the calcium (Ca^2+^) homeostasis and a sustained increase of the Ca^2+^ resting level in response to PAF exposure are directly connected with PAF toxicity in *Neurospora crassa* [8,9].

The cyclic nucleotide cAMP and cellular Ca^2+^, both second messengers, allow the integration of information originating from multiple upstream inputs and enable quick transmission of signals through the cell. The interaction of cAMP mediated signaling and cytoplasmic Ca^2+^ has been intensively studied in animal, plant and yeast cells (reviewed in [10]), whereas the cross-talk and regulation of these pathways is less well understood in filamentous fungi.

PKA consists of two catalytic (PKA_C_) and two regulatory (PKA_R_) subunits forming a heterodimer. Upon activation, four molecules of cAMP bind to the PKA_R_ subunits resulting in the release of PKA_C_, which phosphorylates down-stream targets. The PKA activity regulates most diverse cellular processes, e.g. morphology, hyphal growth, conidiation, virulence, pathogenicity and dimorphic switching [11–13].

Ca^2+^ signaling, on the other hand, is typically based on a fast and transient increase in cytosolic free Ca^2+^ ([Ca^2+^]_c_). The unique signature of the [Ca^2+^]_c_ change determines the specificity in the signaling response that regulates a wide range of processes like hyphal tip growth, branching, differentiation, cell cycle, stress response and virulence [14–16].

So far, we were able to observe the involvement of the cAMP/PKA signaling cascade and the perturbation of the Ca^2+^ homeostasis in response to the antifungal compound PAF as independent events in two different model organisms [7,8], but the direct link between both signaling pathways was still missing. The aim of our study was therefore to investigate the cross-talk between cAMP/PKA signal transduction and the perturbation of the Ca^2+^ homeostasis in response to PAF by using one sensitive model organism.

To achieve our objective we took advantage of the elegant *Aspergillus niger* mutant test system previously characterized and described by [17,18]. These *A. niger* strains lack the catalytic subunit (strain Δ*pka*C) or have a multiple copy integration of the catalytic and the regulatory subunit (mc*pka*CR). The mutant mc*pka*CR contains an equal copy number (10 copies) of the genes *pka*C and *pka*R, which are simultaneously over-expressed. The activity of PKA was reported to be 6 times higher in this mutant than in the wt control (0.6 mU/mg vs. 0.1 mU/ml, respectively). PKA activity is, however, still under the control of PKA_R_ and cAMP [18]. In contrast, the Δ*pka*C has no detectable PKA activity [18]. Importantly, these strains express the codon-optimized Ca^2+^ sensitive photo-protein aequorin for the determination of the [Ca^2+^]_c_ signature in response to external stimuli [15].

Our investigations proved for the first time our hypothesis that cAMP/PKA signaling and the sustained elevation of [Ca^2+^]_c_ in response to PAF treatment are interconnected and regulate PAF toxicity. Our study further underline that this mechanistic function of the antifungal protein PAF is common in sensitive fungi.

## 2 Materials and methods

### 2.1 Strains and chemicals

*A. niger* strains used in this study are listed in Table 1. All strains were obtained from the strain collection of the Department of Biotechnology, National Institute of Chemistry, Ljubljana, Slovenia. Chemicals were purchased from Sigma Aldrich (Austria) unless otherwise stated.

### 2.2 Production of PAF

PAF was purified from the supernatant of a 72 h old liquid shake culture of *P. chrysogenum* Q176 (ATCC 10002) as described previously [8]. For the generation of recombinant PAF (mPAF) and a mutated PAF version (PAF^K35A/K38A^) the *Pichia pastoris* KM71 expression system (Invitrogen, Life Technologies, Austria) was used. Site-directed mutagenesis and cloning were performed as described in [3]. For recombinant expression of mPAF and PAF^K35A/K38A^ the manufacturer’s instruction (Invitrogen) was applied. In brief, one single colony of positively transformed *P. pastoris*, respectively, was used to grow a preculture in 1 L BMG (1.34% YNB, 4 × 10^−5^% biotin, 1% glycerol, 100 mM potassium phosphate pH 6.0) at 28 °C and continuous shaking until log phase was reached (OD_600_ = 2–6). The 1 L preculture was pelleted, resuspended in 100 ml BMM (BMG with 0.5% methanol instead of 1% glycerol) and grown under the same conditions as the preculture, whereby 100% methanol was added to the culture to a final concentration of 0.5% every 24 h. After 96 h of cultivation, the supernatant was collected for protein purification as described in [3].

### 2.3 NMR measurements

To prove the folded structure of PAF^K35A/K38A^, ^1^H NMR was applied. Approximately 0.2 mg protein was dissolved in 450 μl 10 mM sodium phosphate buffer (pH 6.0), then 5% D_2_O was added to the solution which was filled into a 5 mm diameter glass NMR tube. ^1^H NMR spectrum was obtained with water suppression using 3–9–19 pulse sequence with gradients [19]. Since protein concentration was low, 512 scans were needed for a proper spectrum with adequate signal to noise ratio. Two dimensional homonuclear ^1^H–^1^H NOESY spectrum was acquired as well, where watergate W5 pulse sequence water suppression was applied with gradients [20]. Spectra were acquired on a Bruker Avance II 500 MHz spectrometer equipped with a 5 mm Z-gradient triple resonance probe head (Rheinstetten, Germany). Topspin 3.0 software (Bruker GmbH, Rheinstetten, Germany) was used for data acquisition, processing and plotting.

### 2.4 Growth inhibition assays

Antifungal activity assays were performed on appropriately supplemented solid Vogel’s medium containing PAF (0–200 μg/ml) on which 1 × 10^4^ conidia were dotted in 5 μl aliquots. The plates were then incubated at 30 °C for up to 72 h. Every 24 h the plates were photographed using a camera stand with the same fixed distance to the plates. Additionally, the colony diameters were determined. Activity assays with various concentrations of PAF, mPAF and PAF^K35A/K38A^ (0–200 μg/ml) were performed in liquid Vogel’s medium in 96-well plates as described previously [21]. The growth was monitored photometrically at OD_620 nm_ in a microtiter plate reader (GENios Plus, Tecan, Austria) every 24 h and 48 h. All experiments were repeated at least twice.

### 2.5 Measurement of the [Ca^2+^]_c_

*A. niger* strains expressing codon optimized aequorin were inoculated at 1–5 × 10^5^ conidia/ml in Vogel’s medium containing 10 μM coelenterazine (Biosynth, Switzerland) and grown at 30 °C for 12 h in the dark. The calibration of [Ca^2+^]_c_ and the determination of the [Ca^2+^]_c_ signatures were performed as described in [22] using a Microlumat LB96P plate luminometer (Berthold, Germany). All measurements were done in triplicates and repeated at least twice.

## 3 Results and discussion

### 3.1 The lack of the catalytic subunit PKA_C_ renders *A. niger* resistant to PAF

**Fig. 1:**
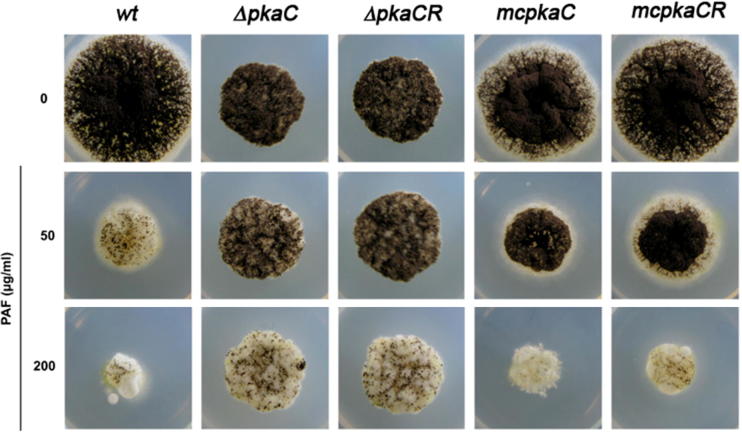
Susceptibility of *A. niger* wt and the PKA mutants Δ*pkaC*, Δ*pkaCR*, mc*pkaC* and mc*pkaCR* to increasing concentrations of PAF. The colony morphology was documented after 72 h of incubation at 30 °C on Vogel’s solid medium.

We exposed the *A. niger* strains with different PKA activities to increasing concentrations of PAF and determined their growth on solid medium. To this end we used the aequorin-expressing mutants Δ*pka*C and mc*pka*CR and included also the strains Δ*pka*CR and mc*pka*C without aequorin expression to investigate the role of the regulatory subunit of PKA in PAF toxicity. Under control conditions (no PAF) the PKA over-expressing mutants, mc*pka*C and mc*pka*CR, showed similar growth and development as the wt, whereas the PKA deletion strains Δ*pka*C and Δ*pka*CR exhibited smaller colony diameters compared to the wt and the mc*pka* mutants (Fig. 1, Table 2). Our observations matched with the phenotype description of these mutants by [18]. However, at a concentration of 200 μg/ml PAF, the Δ*pka*C and Δ*pka*CR strains showed a similar proliferation as the untreated controls, although asexual development was delayed (Fig. 1, Table 2).

In contrast, the radial growth of the wt strain was significantly reduced at a PAF concentration as low as 50 μg/ml (Fig. 1, Table 2). However, the mutants with elevated PKA activity, mc*pka*C and mc*pka*CR, were slightly less sensitive to 50 μg/ml PAF and exhibited enhanced conidiation compared to the wt. Instead, at high PAF concentrations (200 μg/ml), both multi-copy mutants were similarly susceptible to the antifungal protein as the wt, showing reduced colony diameters and delayed conidiation (Fig. 1, Table 2).

Our data therefore indicate that the lack of the catalytic subunit of PKA rescued *A. niger* from PAF toxicity, whereas increased PKA activity did not significantly change the fungal sensitivity to high PAF concentrations (200 μg/ml). Furthermore, neither the additional deletion nor the increased copy number of the regulatory subunits in the mutants, Δ*pka*CR and mc*pka*CR, respectively, resulted in any additional visible effects on the susceptibility of *A. niger* to PAF compared to the single mutants Δ*pka*C and mc*pka*C. This indicates that PKA_C_ plays a dominant role over PKA_R_ in the PAF-specific response. The asexual development of the Δ*pka*C and Δ*pka*CR strains, however, was negatively affected by PAF and seemed not to be under the direct control of PKA activity under the test conditions applied. On the other hand, we have to note here that deregulated expression of PKA may trigger so far undefined rescue mechanisms that result in enhanced conidiation at low PAF concentrations (50 μg/ml) as observed with the mutants mc*pka*C and mc*pka*CR. However, at high PAF concentrations (200 μg/ml) these mechanisms may not be efficient enough to overcome the toxic effect in both mutants.

For completeness we note here that the expression of recombinant aequorin in the *A. niger* strains Δ*pka*C and mc*pka*CR did not influence the sensitivity toward PAF (Fig. 1, Table 2). This was also reflected in a comparable susceptibility of the aequorin-expressing *A. niger* wt and the untransformed wt strain (data not shown).

### 3.2 PAF triggers a specific Ca^2+^ signature in *A. niger*

**Fig. 2:**
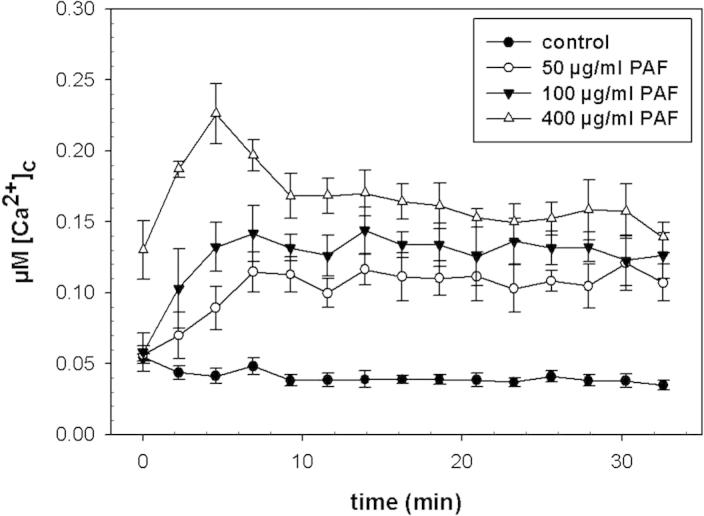
Increase of the [Ca^2+^]_c_ resting level of 12 h-old *A. niger* germlings exposed to 50–400 μg/ml PAF. Samples treated with buffer were used as controls. After the addition of PAF, measurements were taken every 2 min. Values represent the mean of six samples ± S.D.

To characterize the Ca^2+^ response to PAF, we used the aequorin-expressing *A. niger* wt strain. When exposing 12 h old *A. niger* wt germlings to PAF (0–400 μg/ml) a significant, PAF-concentration dependent and sustained elevation of the intracellular Ca^2+^ resting level could be observed (Fig. 2). The [Ca^2+^]_c_ resting level of untreated samples was 0.04 μM (S.D. ⩽ 10%). When applying 400 μg/ml PAF, the [Ca^2+^]_c_ reached 0.23 μM (S.D. ± 0.02) within the first five min before it decreased within the next five min to remain elevated at approx. 0.15 μM (S.D. ⩽ 10%) for the duration of measurement (30 min). Instead, the intracellular Ca^2+^ resting level of the untreated control remained at 0.04 μM (S.D. ⩽ 10%). Notably, we used up to 400 μg/ml PAF to monitor the Ca^2+^ response because fungal germlings are less sensitive to PAF than conidia [8]. To further prove that the Ca^2+^ response is PAF specific, we exposed the wt strain to the protein variant PAF^K35A/K38A^. This recombinant PAF variant carries the exchange of two lysine residues (K) at the positions 35 and 38 of the mature protein for two alanines, respectively, and was expressed in *P. pastoris*. For internal control, a recombinant PAF wt protein (mPAF) was produced in *P. pastoris* as well. In liquid growth inhibition assays mPAF exhibited a similar activity against *A. niger* as PAF, whereas the PAF^K35A/K38A^ variant was significantly less active (Table 3). Notably, the ^1^H NMR and the NOESY spectrum clearly indicated that the PAF^K35A/K38A^ variant was in a folded state (Supplementary data, Fig. S1) and the structure resembled that of PAF. Therefore, unfolding of the PAF variant could be excluded to be responsible for the loss of function of PAF^K35A/K38A^.

Next, we tested the effect of PAF^K35A/K38A^ on the intracellular Ca^2+^ resting level of aequorin expressing *A. niger* germlings. In accordance to our previous observation that PAF toxicity is directly connected with the perturbation of the Ca^2+^ homeostasis, mPAF elicited a similar sustained elevation of the Ca^2+^ resting level as PAF in 12 h *A. niger* germlings (Table 4). In contrast, the PAF^K35A/K38A^ variant failed to trigger this specific response and the [Ca^2+^]_c_ remained at the level of the untreated control sample (Table 4). This result underlines the specificity of the PAF-elicited Ca^2+^ response and gives further evidence that the antifungal toxicity of PAF is directly connected with the perturbation of the fungal Ca^2+^ homeostasis. Furthermore, our data underline our previous suggestion that cationic motifs on the protein surface regulate the interaction of PAF with sensitive target organisms and are directly involved in mediating antifungal toxicity [3].

### 3.3 *A. niger* Δ*pka*C does not respond with a PAF-specific Ca^2+^ elevation

To study the cross-talk between cAMP/PKA signaling and the Ca^2+^ response to PAF, we compared the Ca^2+^ signature in the aequorin-expressing PKA mutants Δ*pka*C and mc*pka*CR with that of the wt. Interestingly, the mutant Δ*pka*C with reduced susceptibility to PAF exhibited a Ca^2+^ resting level 3 times higher than that of the wt strain (approx. 0.14 μM vs. 0.03 μM, respectively). In this mutant, PAF failed to trigger a specific Ca^2+^ response and the [Ca^2+^]_c_ resting level of the treated sample remained at the level of the untreated control (0.14 μM, S.D. ⩽ 10%) for the time of measurement with no significant relative rise in Ca^2+^ (0.7% change to control) (Table 5). In contrast, a significant increase of the Ca^2+^ resting level was triggered by PAF in the PKA over-expressing mutant mc*pka*CR, although the % change was less pronounced than in the wt (+118% vs. +500% change to control, respectively) (Table 5). Nevertheless, we conclude from this finding that antifungal toxicity is mediated by the ability of PAF to evoke a significant elevation of the [Ca^2+^]_c_ resting level.

It had been reported previously, that the Ca^2+^ channel activity is regulated by PKA-dependent phosphorylation [17]. The former characterization of the Δ*pka*C mutant revealed that Ca^2+^ signaling was impaired and the [Ca^2+^]_c_ kinetics in response to mechanical perturbation was significantly reduced [17]. This might explain, why this mutant exhibited an elevated intracellular Ca^2+^ resting level even without PAF challenge and PAF was unable to elicit a specific Ca^2+^ response. However, increased activation of Ca^2+^ channel activity in the mc*pka*CR mutant did not further augment the Ca^2+^ response to PAF. We therefore hypothesize that PAF might itself directly or indirectly interfere with Ca^2+^ channel activity. However, deregulation of the PKA signaling might have other/additional effects on the Ca^2+^ homeostasis and explain the differences in strain-specific susceptibility for PAF: (i) The amount and composition of specific Ca^2+^ channels/pumps/transporters of the PKA mutants might be different to the wt strain, ultimately affecting the dynamics of the Ca^2+^ response. (ii) Jernejc and Benčina [23] demonstrated the PKA-dependent regulation of the lipid biosynthesis in *A. niger*: PKA mutants exhibited differences in the lipid composition. The mutant lacking PKA activity had an increased content of total lipids and a 30% reduction in phospholipids, whereas the mutant with increased PKA activity showed basically a similar lipid content as the wt strain. This was suggested to affect the permeability and fluidity of the plasma membrane and consequently to have impact on the distribution and activity of Ca^2+^ channels/pumps/transporters [23]. Moreover, cell signaling may be affected since phospholipids also cover an important role as second messengers in signal transduction regulating many different cellular processes in response to environmental stimuli [23,24]. (iii) Finally, the activity of another cellular compound may modulate PKA signaling and influence/interfere with the activity of Ca^2+^ channels/pumps/transporters. Some fungi contain two or more catalytic PKA subunits with overlapping or distinct functions [25,26]. In *A. nidulans* PkaB_C_, has a congruent as well as opposite function to PKA_C_ in growth, conidiation and germination depending on the nutrient availability [27]. We found that the *A. niger* genome indeed contains the annotated gene An07g05060 coding for a putative orthologue of the *A. nidulans* PkaB (AN4717), the *Aspergillus fumigatus* PkaC2 (Afu5g08570) and the *Aspergillus oryzae* PkaB (AO090120000207). The function of PkaB in Ca^2+^ signaling, however, has not been characterized so far and awaits detailed investigation in the future.

## 4 Conclusion

This study provides for the first time new insights into the interrelation between Ca^2+^ and cAMP/PKA signaling in *A. niger* in response to the antifungal protein PAF. Our data indicate that the fast and sustained increase of the [Ca^2+^]_c_ resting level is directly linked with PAF toxicity and is specific to a functional protein: the protein variant PAF^K35A/K38A^ is less active and does not elicit a PAF-specific Ca^2+^ signature. Furthermore, activation of the cAMP/PKA signaling cascade is required for PAF activity and is closely connected to Ca^2+^ signaling. The *A. niger* mutant defective in cAMP/PKA signaling and Ca^2+^ response was more resistant toward PAF than the wt strain.

The prominent role of Ca^2+^ signaling in mediating the toxicity of antifungal proteins has been reported in former studies that investigated the mode of action of PAF and the plant defensins MsDef1 and MtDef4 in *N. crassa* [8,28] and the *Aspergillus giganteus* antifungal protein AFP_NN5353_ in *A. niger* [22]. This study underlines the conclusion that the perturbation of the Ca^2+^ homeostasis by antifungal proteins like PAF is a conserved mechanism common to sensitive fungi. Considering the fact that PAF is harmless for mammalian cells, in vitro [29] and in vivo [30], the reported perturbation of the Ca^2+^ homeostasis seems to be specific to fungal cells. The antifungal protein PAF, therefore, represents a promising molecule to develop new antifungal strategies to prevent and combat fungal infections.

## Figures and Tables

**Table 1:** *A. niger* strains used in this study.

Strain	Relevant genotype	Source of reference
wt	Wild-type	CBS 120.49
wt^a^	*cspA1, aeqS, amdS*^+^	[17]
Δ*pka*C^a^	*cspA1, pyrA6, leu A1,* Δ*pkaC::pyrA, nicA1, aeqS*	[17]
Δ*pka*CR	Δ*argB::pyrA1, cspA1, pyrA6, leu A1,* Δ*pkaC::pyrA, nicA1,* Δ*pkaR::argB*	[23]
*mcpka*C	*cspA1, pyrA6, leu A1, nicA1, pkaC*^+^*, pyrA*^+^	[23]
*mcpka*CR^a^	*cspA1, pyrA6, leu A1, nicA1, pkaC*^+^*pkaR*^+^*pyrA*^+^*, aeqS*	[17]

a Aequorin-expressing strains.

**Table 2:** Colony diameters of *A. niger PKA* mutants grown at 30 °C for 72 h on solid Vogel’s medium supplemented with increasing concentrations of PAF (0–200 μg/ml).

PAF [μg/ml]	Strains				
	wt^a^	Δ*pka*C^a^	Δ*pka*CR	mc*pka*C	mc*pka*CR^a^
0	15.0	8.5	10.0	14.0	15.0
50	8.0	8.5	10.0	8.0	10.0
200	5.0	8.5	9.3	5.0	5.0

The values given (in mm) are the mean of three measurements, S.D. < 10%.

a Aequorin-expressing *A. niger* strains.

**Table 3:** The effect of PAF, mPAF and PAF^K35A/K38A^ on the growth of *A. niger*.

Treatment (μg/ml)	% Growth of control (Mean ± S.D.)^a^		
	PAF	mPAF	PAF^K35A/K38A^
1	51.0 ± 4.9	51.0 ± 5.5	106.0 ± 12.4
20	4.6 ± 4.7	0 ± 2.5	78.0 ± 7.7

a The growth was determined by measuring the OD_620 nm_ after 24 h of incubation. The growth of the untreated control cells was normalized to 100% to evaluate the percent growth of samples exposed to the PAF proteins.

**Table 4:** The effect of 400 μg/ml PAF, mPAF and PAF^K35A/K38A^ on the [Ca^2+^]_c_ resting level of aequorin-expressing *A. niger* compared to the untreated controls.

Treatment	Relative rise in [Ca^2+^]_c_ (μM)^a^	% Change^b^
PAF	0.168 ± 0.016	+413
mPAF	0.173 ± 0.012	+428
PAF^K35A/K38A^	0.002 ± 0.004	+3.8

a The relative [Ca^2+^]_c_ rise (measured within 30 ± 5 min) in 12 h old germlings was determined by subtracting the average [Ca^2+^]_c_ of the untreated control from the average [Ca^2+^]_c_ of samples exposed to the respective proteins. Values are means ± S.Ds.

b The average [Ca^2+^]_c_ of the controls was normalized to 100% to evaluate the percent change in [Ca^2+^]_c_ of the treated samples.

**Table 5:** The effect of 400 μg/ml PAF on the relative rise in [Ca^2+^]_c_ on *A. niger* with no detectable PKA activity (Δ*pka*C) and a PkaA over-expressing mutant (mc*pka*CR).

Strains	Relative rise in [Ca^2+^]_c_ (μM)^a^	% Change^b^
*wt*	0.145 ± 0.017	+500
Δ*pkaC*	0.001 ± 0.016	+0.7
mc*pkaCR*	0.055 ± 0.025	+118

a The relative [Ca^2+^]_c_ rise (measured within 30 ± 5 min) in 12 h old germlings was determined by subtracting the average [Ca^2+^]_c_ of the untreated control from the average [Ca^2+^]_c_ of samples exposed to the respective proteins. Values are means ± S.Ds.

b The average [Ca^2+^]_c_ of the controls was normalized to 100% to evaluate the percent change in [Ca^2+^]_c_ of the treated samples.

## Appendix A Supplementary data

Supplementary data This document contains supplementary information.
