# Supplementary material for: Protein kinase A signaling and calcium ions are major players in PAF mediated toxicity against Aspergillus niger
Source: FEBS Lett. 2015 May 8;589(11):1266–71. doi: 10.1016/j.febslet.2015.03.037 (PMC4424949; doi:10.1016/j.febslet.2015.03.037)
Supplement: Supplementary data — This document contains supplementary information. [file mmc1.docx]

**Supplementary Material**

**Figure S1**


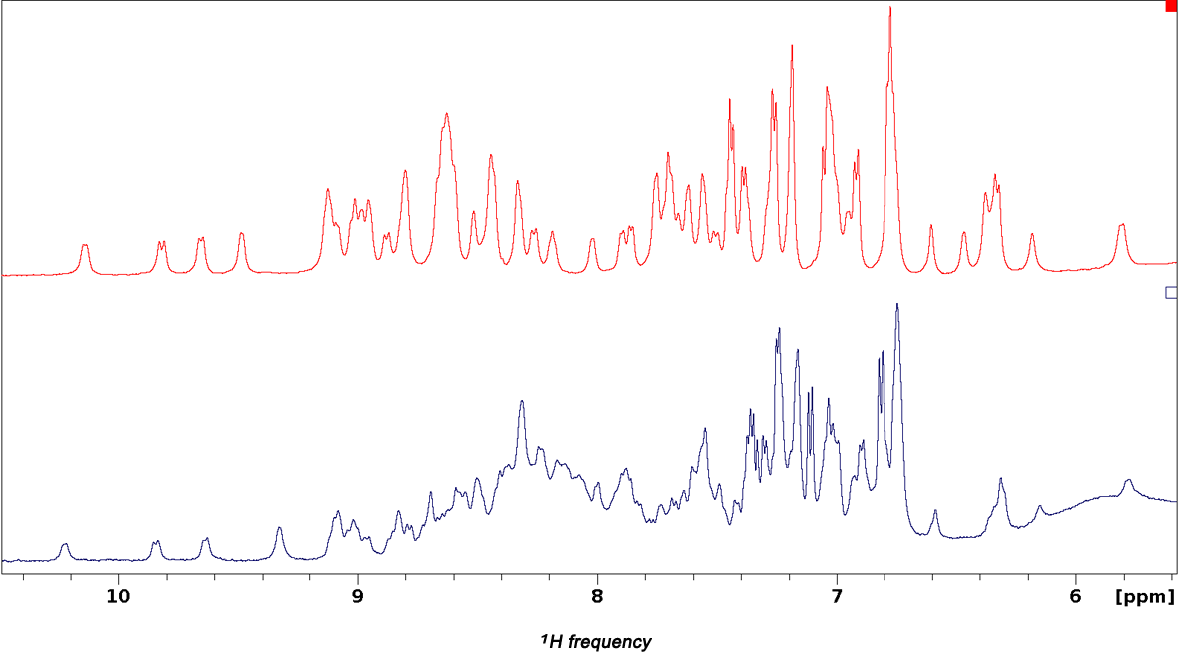


**Figure S1.** Amide and aromatic region of the ^1^H NMR spectrum of native unlabelled PAF (top, red) and PAF^K35A/K38A^ (bottom, blue) in phosphate buffer (pH 6.0). Disperse and narrow ^1^H signals indicate the folded state of both proteins. Similar chemical shifts show the high structural similarity between PAF and PAF^K35A/K38A^.
